# Naphthalene-1,8-dicarb­oxy­lic anhydride: a monoclinic polymorph

**DOI:** 10.1107/S1600536810037608

**Published:** 2010-09-25

**Authors:** Dan Zhao, FeiFei Li, AiYun Zhang

**Affiliations:** a Department of Physics and Chemistry, Henan Polytechnic University, Jiaozuo, Henan 454000, People’s Republic of China

## Abstract

A new type of naphthalene-1,8-dicarb­oxy­lic anhydride, C_12_H_6_O_3_, was synthesized hydro­thermally. Unlike the two previously reported polymorphs, which crystallize in the ortho­rhom­bic space groups *P*2_1_2_1_2_1_ [Shok *et al.* (1971). *Kristallografiya*, **16**, 500–502; Grigor’eva & Chetkina (1975). *Kristallografiya*, **20**, 1289–1290] and *Pbca* [Shok & Gol’der (1970). *Zh. Strukt. Khim.* **11**, 939–940], this present structure crystallizes in the monoclinic space group *P*2_1_ */c*. In this structure, the planar [total puckering amplitude *Q* = 0.0362 (15)] mol­ecules lie parallel to each other along the *a* axis.

## Related literature

The previously reported polymorphs crystallize in *P*2_1_2_1_2_1_ (Shok *et al.*, 1971 ▶; Grigor’eva & Chetkina, 1975 ▶) and *Pbca* (Shok & Gol’der, 1970 ▶). For puckering parameters, see: Evans & Boeyens (1989 ▶).
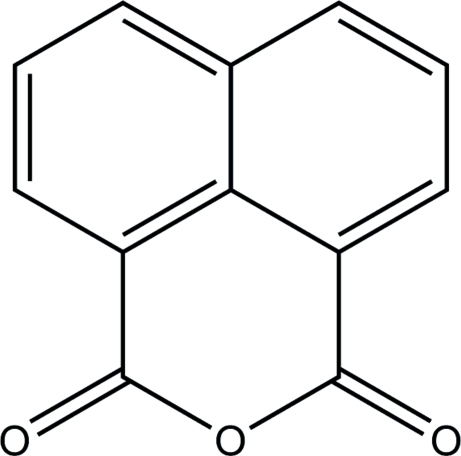


## Experimental

### 

#### Crystal data

C_12_H_6_O_3_
*M* *_r_* = 198.17
Monoclinic, 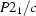

*a* = 3.7687 (1) Å
*b* = 14.5269 (3) Å
*c* = 15.8083 (3) Å
β = 94.752 (2)°
*V* = 862.49 (3) Å^3^
*Z* = 4
Mo *K*α radiation
μ = 0.11 mm^−1^
*T* = 296 K
0.20 × 0.10 × 0.10 mm

#### Data collection

Bruker APEXII CCD diffractometer
Absorption correction: multi-scan (*SADABS*; Sheldrick, 1996 ▶) *T*_min_ = 0.875, *T*_max_ = 0.982
7560 measured reflections
1964 independent reflections
1201 reflections with *I* > 2σ(*I*)
*R*_int_ = 0.027

#### Refinement

*R*[*F* ^2^ > 2σ(*F* ^2^)] = 0.045
*wR*(*F* ^2^) = 0.131
*S* = 1.00
1964 reflections
136 parameters
H-atom parameters constrained
Δρ_max_ = 0.18 e Å^−3^
Δρ_min_ = −0.17 e Å^−3^

### 

Data collection: *APEX2* (Bruker, 2007 ▶); cell refinement: *SAINT* (Bruker, 2007 ▶); data reduction: *SAINT*; program(s) used to solve structure: *SHELXS97* (Sheldrick, 2008 ▶); program(s) used to refine structure: *SHELXL97* (Sheldrick, 2008 ▶); molecular graphics: *SHELXTL* (Sheldrick, 2008 ▶); software used to prepare material for publication: *SHELXTL*.

## Supplementary Material

Crystal structure: contains datablocks I, global. DOI: 10.1107/S1600536810037608/hg2709sup1.cif

Structure factors: contains datablocks I. DOI: 10.1107/S1600536810037608/hg2709Isup2.hkl

Additional supplementary materials:  crystallographic information; 3D view; checkCIF report

## Figures and Tables

**Fig. 1.**
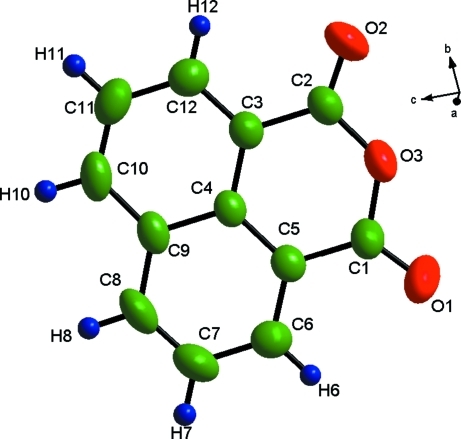
The molecular structure of the title compound. Displacement ellipsoids are drawn at the 50% probability level. H atoms are omitted for clarity. [Symmetry code: x, y, z]

**Fig. 2.**
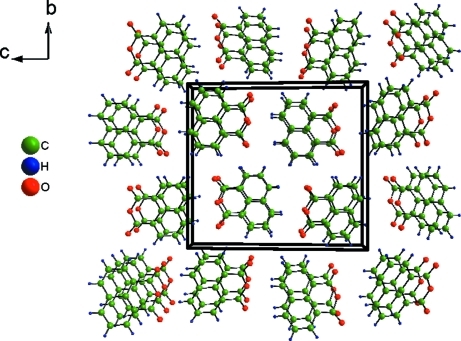
A packing diagram of the title compound viewed down the a–axis.

## supplementary crystallographic information

### Comment

1,8-Naphthalenedicarboxylate (1,8-NDC), can be used as a rigid building blocks to design multiple metal-organic coordination polymers, as its multiple coordination sites, high symmetry and large conjugated structure. The single-crystal structure of naphthalene-1,8-dicarboxylic anhydride was firstly determined by Shok and Gol'der to be a orthorhombic space group *Pbca* (Shok, *et al.*, 1970). Later a β-phase was discovered with the space group *P*2_1_2_1_2_1_ (Shok *et al.*, 1971; Grigor'eva & Chetkina, 1975). In this paper, a new type of naphthalene-1,8-dicarboxylic acid anhydride was hydrothermally synthesized and characterized by single-crystal X-ray diffraction with the monoclinic space group *P*2_1_/*c*.

The asymmetric unit contains only one independent molecule with the planar [total puckering amplitude Q = 0.0362 (15) (Evans & Boeyens, 1989)] molecules parallel to each other along the *a*-axis (Fig. 2).

### Experimental

Yellow prism-shaped single crystals of Naphthalene-1,8-dicarboxylic acid anhydride were initially obtained in our attempt to prepare metal-organic coordination polymers of 1,8-NDC associated with molybdate. A mixture of 3 mmol of MoO_3_, 2 mmol of Mn(Ac)_2_, 2.0 mmol KOH and 1.5 mmol of Naphthalene-1,8-dicarboxylic anhydride, was sealed in a 25 ml Teflonlined bomb at 160°C for 5 days and then cooled to room temperature. A few single crystals suitable for X-ray diffraction analysis were obtained.

### Refinement

All of the H atoms were treated as riding atoms with distances C—H = 0.93 Å (CH), and *U*_iso_(H) = 1.2 *U*_eq_(C). The final refinement show that the highest peak in the difference electron density map equals to 0.18 e/Å^3^ at the distance of 0.65 Å from C5 while the deepest hole equals to -0.17 e/Å^3^ at the distance of 0.61 Å from C1.

### Crystal data

**Table:**

C_12_H_6_O_3_	*F*(000) = 408
*M**_r_* = 198.17	*D*_x_ = 1.526 Mg m^−^^3^
Monoclinic, *P*2_1_/*c*	Mo *K*α radiation, λ = 0.71073 Å
Hall symbol: -P 2ybc	Cell parameters from 1938 reflections
*a* = 3.7687 (1) Å	θ = 2.6–27.9°
*b* = 14.5269 (3) Å	µ = 0.11 mm^−^^1^
*c* = 15.8083 (3) Å	*T* = 296 K
β = 94.752 (2)°	Prism, yellow
*V* = 862.49 (3) Å^3^	0.20 × 0.10 × 0.10 mm
*Z* = 4	

### Data collection

**Table:**

Bruker APEXII CCD diffractometer	1964 independent reflections
Radiation source: fine-focus sealed tube	1201 reflections with *I* > 2σ(*I*)
graphite	*R*_int_ = 0.027
ω scans	θ_max_ = 27.5°, θ_min_ = 1.9°
Absorption correction: multi-scan (*SADABS*; Sheldrick, 1996)	*h* = −4→4
*T*_min_ = 0.875, *T*_max_ = 0.982	*k* = −17→18
7560 measured reflections	*l* = −20→20

### Refinement

**Table:**

Refinement on *F*^2^	Primary atom site location: structure-invariant direct methods
Least-squares matrix: full	Secondary atom site location: difference Fourier map
*R*[*F*^2^ > 2σ(*F*^2^)] = 0.045	Hydrogen site location: inferred from neighbouring sites
*wR*(*F*^2^) = 0.131	H-atom parameters constrained
*S* = 1.00	*w* = 1/[σ^2^(*F*_o_^2^) + (0.0732*P*)^2^] where *P* = (*F*_o_^2^ + 2*F*_c_^2^)/3
1964 reflections	(Δ/σ)_max_ < 0.001
136 parameters	Δρ_max_ = 0.18 e Å^−^^3^
0 restraints	Δρ_min_ = −0.17 e Å^−^^3^

### Special details

**Table:**

Geometry. All e.s.d.'s (except the e.s.d. in the dihedral angle between two l.s. planes) are estimated using the full covariance matrix. The cell e.s.d.'s are taken into account individually in the estimation of e.s.d.'s in distances, angles and torsion angles; correlations between e.s.d.'s in cell parameters are only used when they are defined by crystal symmetry. An approximate (isotropic) treatment of cell e.s.d.'s is used for estimating e.s.d.'s involving l.s. planes.
Refinement. Refinement of *F*^2^ against ALL reflections. The weighted *R*-factor *wR* and goodness of fit *S* are based on *F*^2^, conventional *R*-factors *R* are based on *F*, with *F* set to zero for negative *F*^2^. The threshold expression of *F*^2^ > σ(*F*^2^) is used only for calculating *R*-factors(gt) *etc*. and is not relevant to the choice of reflections for refinement. *R*-factors based on *F*^2^ are statistically about twice as large as those based on *F*, and *R*- factors based on ALL data will be even larger.

### Fractional atomic coordinates and isotropic or equivalent isotropic displacement parameters (Å^2^)

**Table:**

	*x*	*y*	*z*	*U*_iso_*/*U*_eq_	
C5	0.3898 (4)	0.64036 (9)	0.28567 (8)	0.0399 (4)	
C1	0.2469 (4)	0.64100 (10)	0.19687 (9)	0.0486 (4)	
C6	0.5444 (4)	0.56205 (10)	0.32018 (9)	0.0503 (4)	
H6	0.5618	0.5098	0.2868	0.060*	
C11	0.3060 (5)	0.87580 (12)	0.43612 (10)	0.0588 (5)	
H11	0.2878	0.9279	0.4696	0.071*	
C4	0.3603 (3)	0.72008 (9)	0.33538 (8)	0.0356 (4)	
C3	0.1982 (4)	0.80075 (10)	0.30145 (8)	0.0397 (4)	
C2	0.0514 (4)	0.80154 (11)	0.21286 (9)	0.0478 (4)	
C8	0.6540 (4)	0.63636 (11)	0.45452 (10)	0.0544 (5)	
H8	0.7441	0.6341	0.5111	0.065*	
C7	0.6755 (4)	0.56069 (12)	0.40523 (10)	0.0571 (5)	
H7	0.7788	0.5072	0.4284	0.068*	
C9	0.4972 (4)	0.71882 (10)	0.42179 (8)	0.0429 (4)	
O3	0.0835 (3)	0.72159 (7)	0.16646 (6)	0.0557 (3)	
C12	0.1693 (4)	0.87738 (11)	0.35104 (9)	0.0504 (4)	
H12	0.0594	0.9302	0.3283	0.061*	
C10	0.4642 (4)	0.79925 (12)	0.47005 (9)	0.0555 (4)	
H10	0.5534	0.7998	0.5267	0.067*	
O2	−0.1003 (4)	0.86395 (8)	0.17671 (7)	0.0747 (4)	
O1	0.2558 (4)	0.57887 (8)	0.14772 (7)	0.0766 (4)	

### Atomic displacement parameters (Å^2^)

**Table:**

	*U*^11^	*U*^22^	*U*^33^	*U*^12^	*U*^13^	*U*^23^
C5	0.0368 (8)	0.0459 (9)	0.0377 (8)	−0.0059 (7)	0.0073 (6)	0.0044 (6)
C1	0.0597 (10)	0.0486 (9)	0.0376 (8)	−0.0111 (8)	0.0043 (7)	0.0002 (7)
C6	0.0511 (10)	0.0467 (9)	0.0544 (10)	−0.0002 (7)	0.0116 (8)	0.0037 (7)
C11	0.0618 (11)	0.0624 (11)	0.0531 (10)	−0.0070 (9)	0.0104 (8)	−0.0178 (9)
C4	0.0306 (7)	0.0447 (8)	0.0321 (7)	−0.0061 (6)	0.0063 (6)	0.0035 (6)
C3	0.0357 (8)	0.0463 (8)	0.0376 (8)	−0.0043 (7)	0.0053 (6)	0.0023 (7)
C2	0.0499 (9)	0.0539 (9)	0.0391 (8)	0.0002 (8)	0.0008 (7)	0.0061 (7)
C8	0.0447 (10)	0.0789 (12)	0.0387 (8)	−0.0011 (8)	−0.0020 (7)	0.0195 (9)
C7	0.0506 (11)	0.0605 (11)	0.0600 (10)	0.0071 (8)	0.0043 (8)	0.0210 (9)
C9	0.0345 (8)	0.0618 (10)	0.0325 (7)	−0.0063 (7)	0.0033 (6)	0.0027 (7)
O3	0.0705 (8)	0.0593 (7)	0.0354 (6)	−0.0055 (6)	−0.0075 (5)	0.0030 (5)
C12	0.0495 (10)	0.0483 (9)	0.0545 (10)	0.0006 (7)	0.0103 (8)	0.0005 (7)
C10	0.0524 (10)	0.0797 (12)	0.0339 (8)	−0.0086 (9)	0.0014 (7)	−0.0075 (8)
O2	0.0918 (10)	0.0725 (8)	0.0572 (7)	0.0227 (7)	−0.0100 (7)	0.0185 (6)
O1	0.1232 (12)	0.0586 (8)	0.0480 (7)	−0.0138 (7)	0.0075 (7)	−0.0131 (6)

### Geometric parameters (Å, °)

**Table:**

C5—C6	1.3706 (19)	C3—C12	1.3710 (19)
C5—C4	1.4090 (18)	C3—C2	1.463 (2)
C5—C1	1.4613 (19)	C2—O2	1.1922 (17)
C1—O1	1.1931 (16)	C2—O3	1.3844 (17)
C1—O3	1.3900 (17)	C8—C7	1.354 (2)
C6—C7	1.394 (2)	C8—C9	1.4146 (19)
C6—H6	0.9300	C8—H8	0.9300
C11—C10	1.351 (2)	C7—H7	0.9300
C11—C12	1.400 (2)	C9—C10	1.407 (2)
C11—H11	0.9300	C12—H12	0.9300
C4—C3	1.4072 (18)	C10—H10	0.9300
C4—C9	1.4197 (19)		
			
C6—C5—C4	120.72 (13)	O2—C2—C3	126.39 (15)
C6—C5—C1	119.88 (13)	O3—C2—C3	117.26 (13)
C4—C5—C1	119.39 (12)	C7—C8—C9	121.35 (14)
O1—C1—O3	116.57 (13)	C7—C8—H8	119.3
O1—C1—C5	126.36 (15)	C9—C8—H8	119.3
O3—C1—C5	117.07 (12)	C8—C7—C6	120.70 (14)
C5—C6—C7	120.05 (14)	C8—C7—H7	119.7
C5—C6—H6	120.0	C6—C7—H7	119.7
C7—C6—H6	120.0	C10—C9—C8	123.93 (13)
C10—C11—C12	120.65 (14)	C10—C9—C4	118.02 (13)
C10—C11—H11	119.7	C8—C9—C4	118.05 (13)
C12—C11—H11	119.7	C2—O3—C1	125.38 (11)
C3—C4—C5	121.58 (12)	C3—C12—C11	119.71 (14)
C3—C4—C9	119.28 (12)	C3—C12—H12	120.1
C5—C4—C9	119.13 (12)	C11—C12—H12	120.1
C12—C3—C4	120.72 (12)	C11—C10—C9	121.62 (14)
C12—C3—C2	119.96 (13)	C11—C10—H10	119.2
C4—C3—C2	119.30 (13)	C9—C10—H10	119.2
O2—C2—O3	116.33 (13)		
